# Errors

**Published:** 1912-09

**Authors:** 


### Errors.

Our attention has been called to some errors in the August issue. We are obliged for the criticism but they had already been noticed and made the subject of prayer—at least the individual words might have been in a prayer of the highly orthodox. We would like to make this general announcement as to verbal errors. Contributors are expected to prepare their own articles and to revise proof. We are glad to make an exception in favor of those unfamiliar with the language; otherwise, it seems to us that a grown man ought to be able to dress his thoughts in words just as he is expected to be able to dress his body in clothes. Hence, with occasional exceptions due to distance, etc., hold the author responsible for errors in the original department. In the editorial and various news departments, errors should be charged to the editorial staff. When, as in the August issue, whole lines were dropped or misplaced, it may be taken for granted that both galley and page proof had been properly revised but that a form ready for the press was accidentally dropped and the linotype pi was not properly rearranged. This kind of blunder is about the only one that can properly be charge to the printer and not so much to the compositor as to the men who finally handle the forms. We have had articles in writing which were in parts, unintelligible both to the printer and the editor, so that phrases had to be omitted altogether. Printers such as work on this JOURNAL make surprisingly few mistakes, even in dealing with highly technical matter and we do not believe in making them the scape goat for our own and other writers' blunders.

Dr. W. A. MacPherson of N. Tonawanda, asks us to correct the statements that his patient gained 95 pounds in four or five weeks under Dr. Dowd's phorphorus tonic. This would be too high praise and would produce an idea that this excellent remedy was being exploited by extravagant methods. The fact was that the patient gained 15 pounds. This error was neither due to printer nor proof reader but to the typewriter who prepared the report.

Back Numbers. Owing to a considerable and growing demand for extra copies of the Journal, we would ask subscribers whose copies are lost in the mail or who wish additional copies, to notify us before the tenth of the month. We still have extra copies of most of the issues up to the end of Vol. 66 (1910-11). Of Vol. 67, (1911-12) there are, at the date of writing, just 40 copies in all, an average of less than four an issue, and many of these are marked in various ways; although we have always ordered what seemed to be a liberal number of copies beyond the regular subscription list. This is a wholesome kind of poverty.

---

Dr. Hektoen's lectures will be continued in the October, and concluded in the November issue.

---

When the advertising pages, inclusive of table of contents, a reasonable allowance of "readers," etc., are numbered up to L (50), and can be maintained at that number, the subscription rate will be reduced to $1.50 net, in advance, for subsequent volumes. We ask our readers' cooperation to secure this desired object, both by hints, to us, an occasional word of commendation to prospective advertisers and by paying attention to the advertisements. In corresponding with advertisers, please mention the Journal as any error in following a key will deprive the Journal of the credit due. Also, help all periodicals, the postman and save your own time and that of busy men generally by passing circulars promptly to the W. P. B.

